# Supplementary material for: The Role of RKIP in the Regulation of EMT in the Tumor Microenvironment
Source: Cancers (Basel). 2022 Sep 22;14(19):4596. doi: 10.3390/cancers14194596 (PMC9559516; doi:10.3390/cancers14194596)
Supplement: Supplementary file 1 [file cancers-14-04596-s001.zip › cancers-1924584-supplementary.pdf]

Supplementary Materials

Gene expression (log2(TPM)) of the genes PEBP1 (RKIP), SNAI1 (SNAIL), VIM, CDH1 (E-Cadherin), CDH2 (N-Cadherin), EPCAM, LAMA1 and LAMB1, across 22 different types of cancer in the Cancer Genome Atlas (TCGA). BLCA, Bladder Urothelial Carcinoma; BRCA, Breast invasive carcinoma; CESC, Cervical squamous cell carcinoma and endocervical adenocarcinoma; CHOL, Cholangiocarcinoma; COAD, Colon adenocarcinoma; ESCA, Esophageal carcinoma; HNSC, Head and Neck squamous cell carcinoma; KICH, Kidney Chromophobe; KIRC, Kidney renal clear cell carcinoma; KIRP, Kidney renal papillary cell carcinoma; LIHC, Liver hepatocellular carcinoma; LUAD, Lung adenocarcinoma; LUSC, Lung squamous cell carcinoma; PAAD, Pancreatic adenocarcinoma; PCPG, Pheochromocytoma and Paraganglioma; PRAD, Prostate adenocarcinoma; READ, Rectum adenocarcinoma; SARC, Sarcoma; SKCM, Skin Cutaneous Melanoma; STAD, Stomach adenocarcinoma; THCA, Thyroid carcinoma; THYM, Thymoma; UCEC. Pair-wise Pearson's correlation between PEBP1 and each of the biomarkers (SNAI1, VIM, CDH1, CDH2, EPCAM, LAMA1 and LAMB1) were across for each of the 22 different types of cancer and their normal tissues in the TCGA. Significance is listed for each cancer type, for each biomarker with PEBP1.

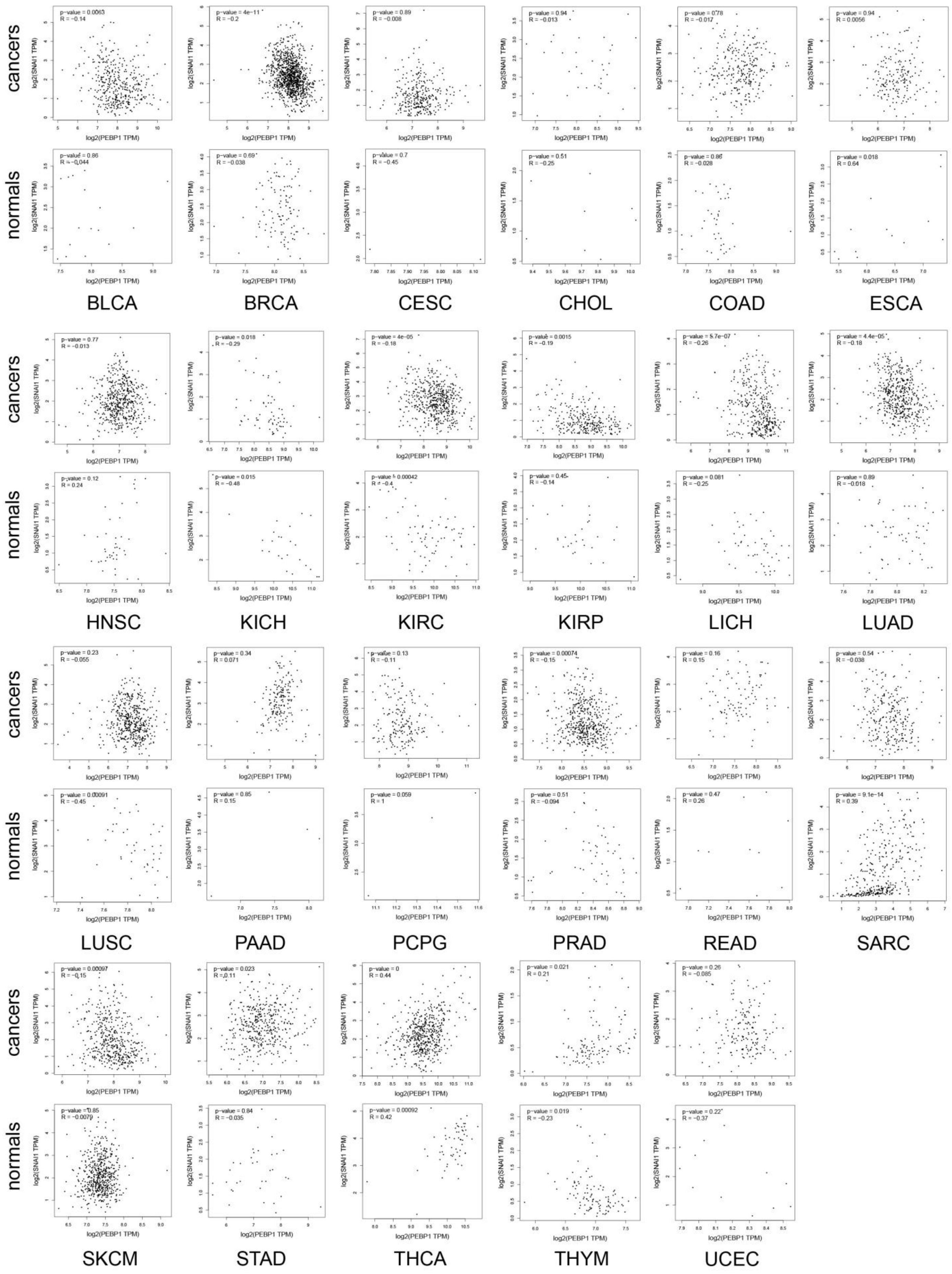
cancers
normals
BLCA
BRCA
CESC
CHOL
COAD
ESCA
HNSC
KICH
KIRC
KIRP
LICH
LUAD
LUSC
PAAD
PCPG
PRAD
READ
SARC
SKCM
STAD
THCA
THYM
UCEC
log2(SNAI1 TPM)
log2(PEBP1 TPM)

Supplementary Figure S1. RKIP vs SNAI1 across normal and cancerous tissues.

Gene expression (log_2(TPM)) of the genes PEBP1 (RKIP), SNAI1 (SNAIL), VIM, CDH1 (E-Cadherin), CDH2 (N-Cadherin), EPCAM, LAMA1 and LAMB1, across 22 different types of cancer in the Cancer Genome Atlas (TCGA). BLCA, Bladder Urothelial Carcinoma; BRCA, Breast invasive carcinoma; CESC, Cervical squamous cell carcinoma and endocervical adenocarcinoma; CHOL, Cholangiocarcinoma; COAD, Colon adenocarcinoma; ESCA, Esophageal carcinoma; HNSC, Head and Neck squamous cell carcinoma; KICH, Kidney Chromophobe; KIRC, Kidney renal clear cell carcinoma; KIRP, Kidney renal papillary cell carcinoma; LIHC, Liver hepatocellular carcinoma; LUAD, Lung adenocarcinoma; LUSC, Lung squamous cell carcinoma; PAAD, Pancreatic adenocarcinoma; PCPG, Pheochromocytoma and Paraganglioma; PRAD, Prostate adenocarcinoma; READ, Rectum adenocarcinoma; SARC, Sarcoma; SKCM, Skin Cutaneous Melanoma; STAD, Stomach adenocarcinoma; THCA, Thyroid carcinoma; THYM, Thymoma; UCEC. Pair-wise Pearson's correlation between PEBP1 and SNAI1 were across for each of the 22 different types of cancer and their normal tissues in the TCGA. Significance is listed for each cancer type, for each biomarker with PEBP1.

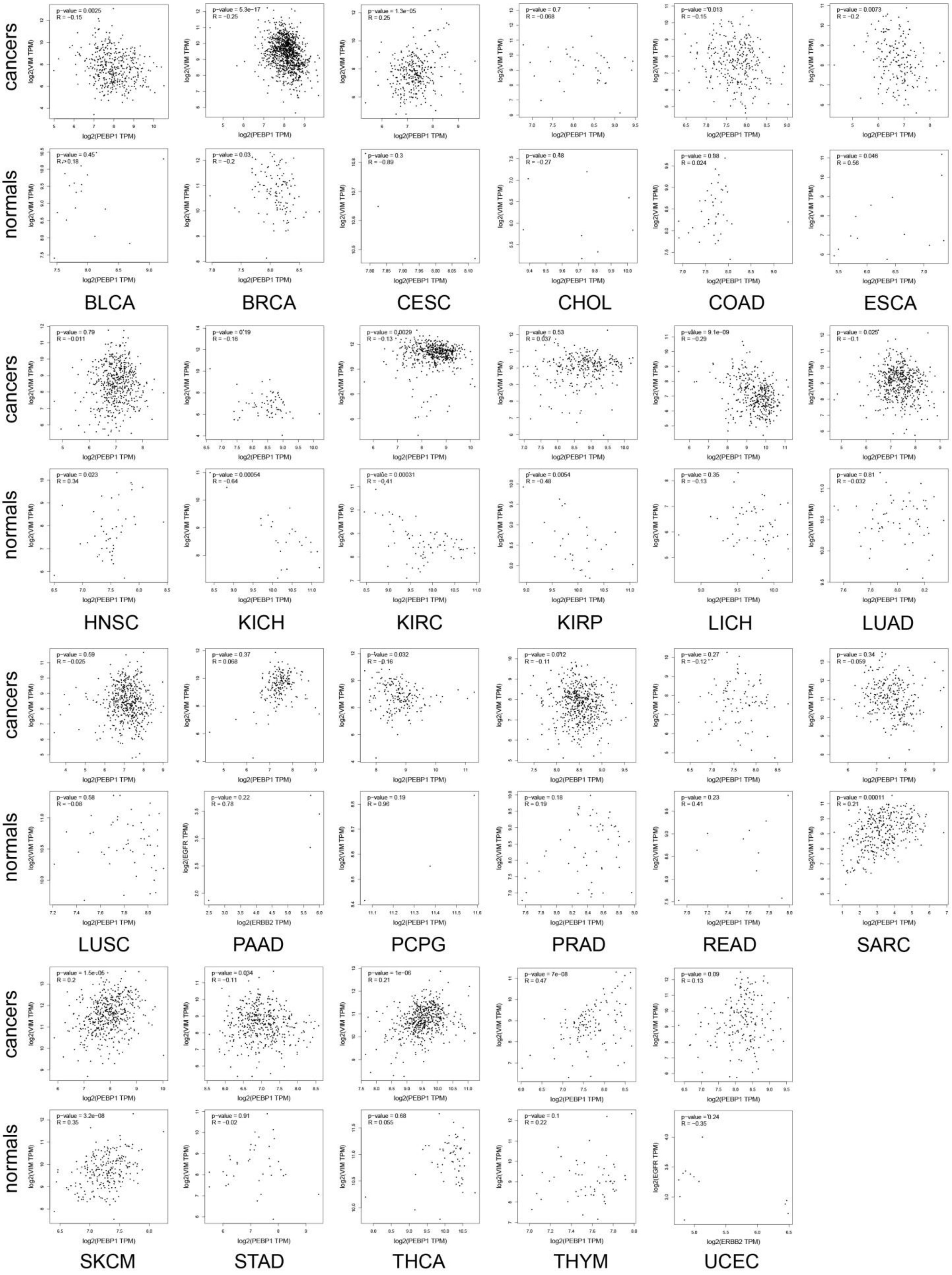
cancers
normals
BLCA
BRCA
CESC
CHOL
COAD
ESCA
HNSC
KICH
KIRC
KIRP
LICH
LUAD
LUSC
PAAD
PCPG
PRAD
READ
SARC
SKCM
STAD
THCA
THYM
UCEC
log2(PEBP1 TPM)
log2(VIM TPM)
log2(EGFR TPM)
log2(ERBB2 TPM)

Supplementary Figure S2. RKIP vs VIM across normal and cancerous tissues.

Gene expression (log_2(TPM)) of the genes PEBP1 (RKIP), SNAI1 (SNAIL), VIM, CDH1 (E-Cadherin), CDH2 (N-Cadherin), EPCAM, LAMA1 and LAMB1, across 22 different types of cancer in the Cancer Genome Atlas (TCGA). BLCA, Bladder Urothelial Carcinoma; BRCA, Breast invasive carcinoma; CESC, Cervical squamous cell carcinoma and endocervical adenocarcinoma; CHOL, Cholangiocarcinoma; COAD, Colon adenocarcinoma; ESCA, Esophageal carcinoma; HNSC, Head and Neck squamous cell carcinoma; KICH, Kidney Chromophobe; KIRC, Kidney renal clear cell carcinoma; KIRP, Kidney renal papillary cell carcinoma; LIHC, Liver hepatocellular carcinoma; LUAD, Lung adenocarcinoma; LUSC, Lung squamous cell carcinoma; PAAD, Pancreatic adenocarcinoma; PCPG, Pheochromocytoma and Paraganglioma; PRAD, Prostate adenocarcinoma; READ, Rectum adenocarcinoma; SARC, Sarcoma; SKCM, Skin Cutaneous Melanoma; STAD, Stomach adenocarcinoma; THCA, Thyroid carcinoma; THYM, Thymoma; UCEC. Pair-wise Pearson's correlation between PEBP1 and VIM were across for each of the 22 different types of cancer and their normal tissues in the TCGA. Significance is listed for each cancer type, for each biomarker with PEBP1.

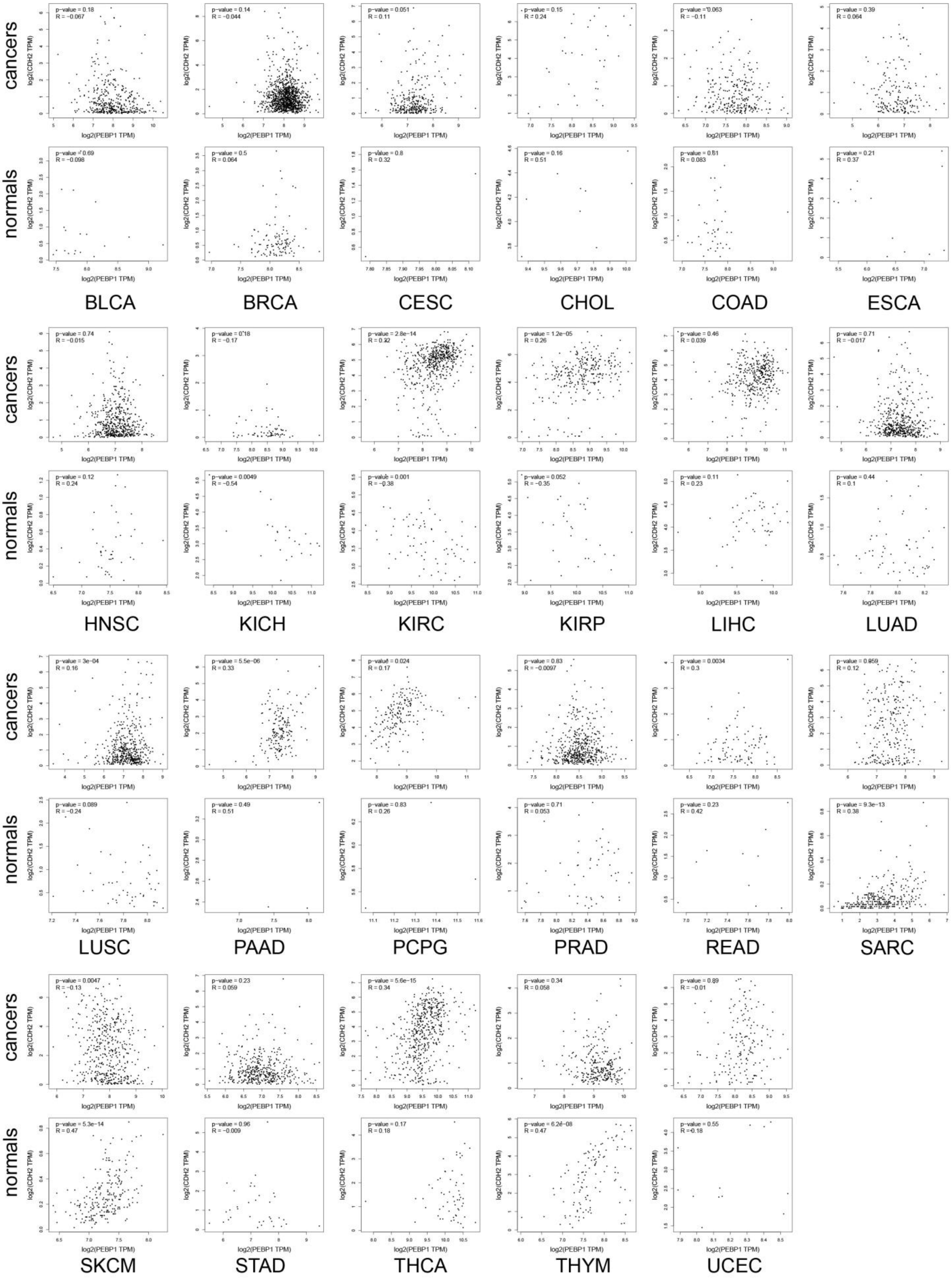

cancers
normals
log2(CDH2 TPM)
log2(PEBP1 TPM)
BLCA
BRCA
CESC
CHOL
COAD
ESCA
HNSC
KICH
KIRC
KIRP
LIHC
LUAD
LUSC
PAAD
PCPG
PRAD
READ
SARC
SKCM
STAD
THCA
THYM
UCEC

Supplementary Figure S3. RKIP vs CDH2 across normal and cancerous tissues.

Gene expression (log_2(TPM)) of the genes PEBP1 (RKIP), SNAI1 (SNAIL), VIM, CDH1 (E-Cadherin), CDH2 (N-Cadherin), EPCAM, LAMA1 and LAMB1, across 22 different types of cancer in the Cancer Genome Atlas (TCGA). BLCA, Bladder Urothelial Carcinoma; BRCA, Breast invasive carcinoma; CESC, Cervical squamous cell carcinoma and endocervical adenocarcinoma; CHOL, Cholangiocarcinoma; COAD, Colon adenocarcinoma; ESCA, Esophageal carcinoma; HNSC, Head and Neck squamous cell carcinoma; KICH, Kidney Chromophobe; KIRC, Kidney renal clear cell carcinoma; KIRP, Kidney renal papillary cell carcinoma; LIHC, Liver hepatocellular carcinoma; LUAD, Lung adenocarcinoma; LUSC, Lung squamous cell carcinoma; PAAD, Pancreatic adenocarcinoma; PCPG, Pheochromocytoma and Paraganglioma; PRAD, Prostate adenocarcinoma; READ, Rectum adenocarcinoma; SARC, Sarcoma; SKCM, Skin Cutaneous Melanoma; STAD, Stomach adenocarcinoma; THCA, Thyroid carcinoma; THYM, Thymoma; UCEC. Pair-wise Pearson's correlation between PEBP1 and CDH2 were across for each of the 22 different types of cancer and their normal tissues in the TCGA. Significance is listed for each cancer type, for each biomarker with PEBP1.

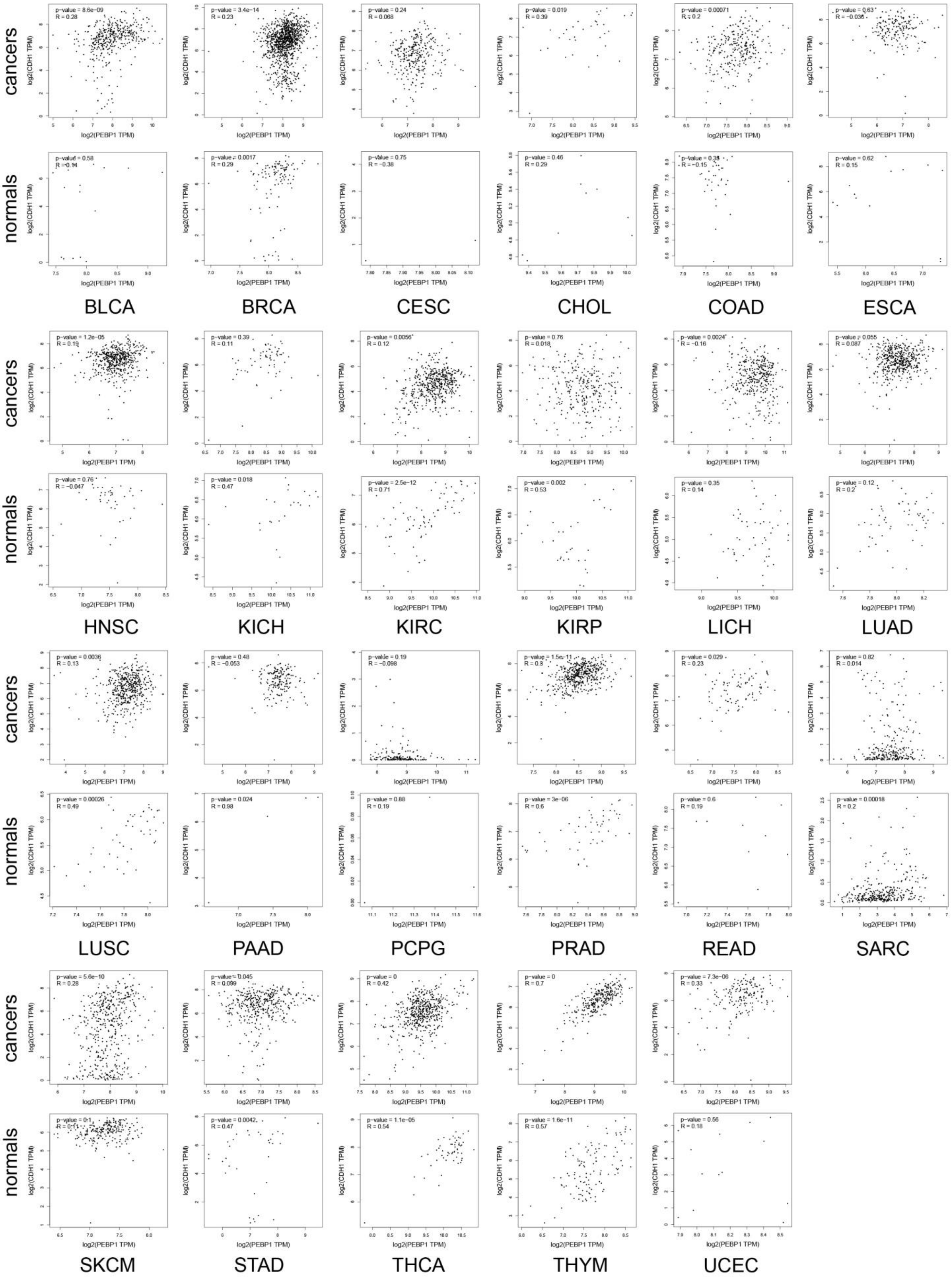
cancers
normals
BLCA
BRCA
CESC
CHOL
COAD
ESCA
HNSC
KICH
KIRC
KIRP
LICH
LUAD
LUSC
PAAD
PCPG
PRAD
READ
SARC
SKCM
STAD
THCA
THYM
UCEC
log2(CDH1 TPM)
log2(PEBP1 TPM)

Supplementary Figure S4. RKIP vs CDH1 across normal and cancerous tissues.

Gene expression (log_2(TPM)) of the genes PEBP1 (RKIP), SNAI1 (SNAIL), VIM, CDH1 (E-Cadherin), CDH2 (N-Cadherin), EPCAM, LAMA1 and LAMB1, across 22 different types of cancer in the Cancer Genome Atlas (TCGA). BLCA, Bladder Urothelial Carcinoma; BRCA, Breast invasive carcinoma; CESC, Cervical squamous cell carcinoma and endocervical adenocarcinoma; CHOL, Cholangiocarcinoma; COAD, Colon adenocarcinoma; ESCA, Esophageal carcinoma; HNSC, Head and Neck squamous cell carcinoma; KICH, Kidney Chromophobe; KIRC, Kidney renal clear cell carcinoma; KIRP, Kidney renal papillary cell carcinoma; LIHC, Liver hepatocellular carcinoma; LUAD, Lung adenocarcinoma; LUSC, Lung squamous cell carcinoma; PAAD, Pancreatic adenocarcinoma; PCPG, Pheochromocytoma and Paraganglioma; PRAD, Prostate adenocarcinoma; READ, Rectum adenocarcinoma; SARC, Sarcoma; SKCM, Skin Cutaneous Melanoma; STAD, Stomach adenocarcinoma; THCA, Thyroid carcinoma; THYM, Thymoma; UCEC. Pair-wise Pearson's correlation between PEBP1 and CDH1 were across for each of the 22 different types of cancer and their normal tissues in the TCGA. Significance is listed for each cancer type, for each biomarker with PEBP1.

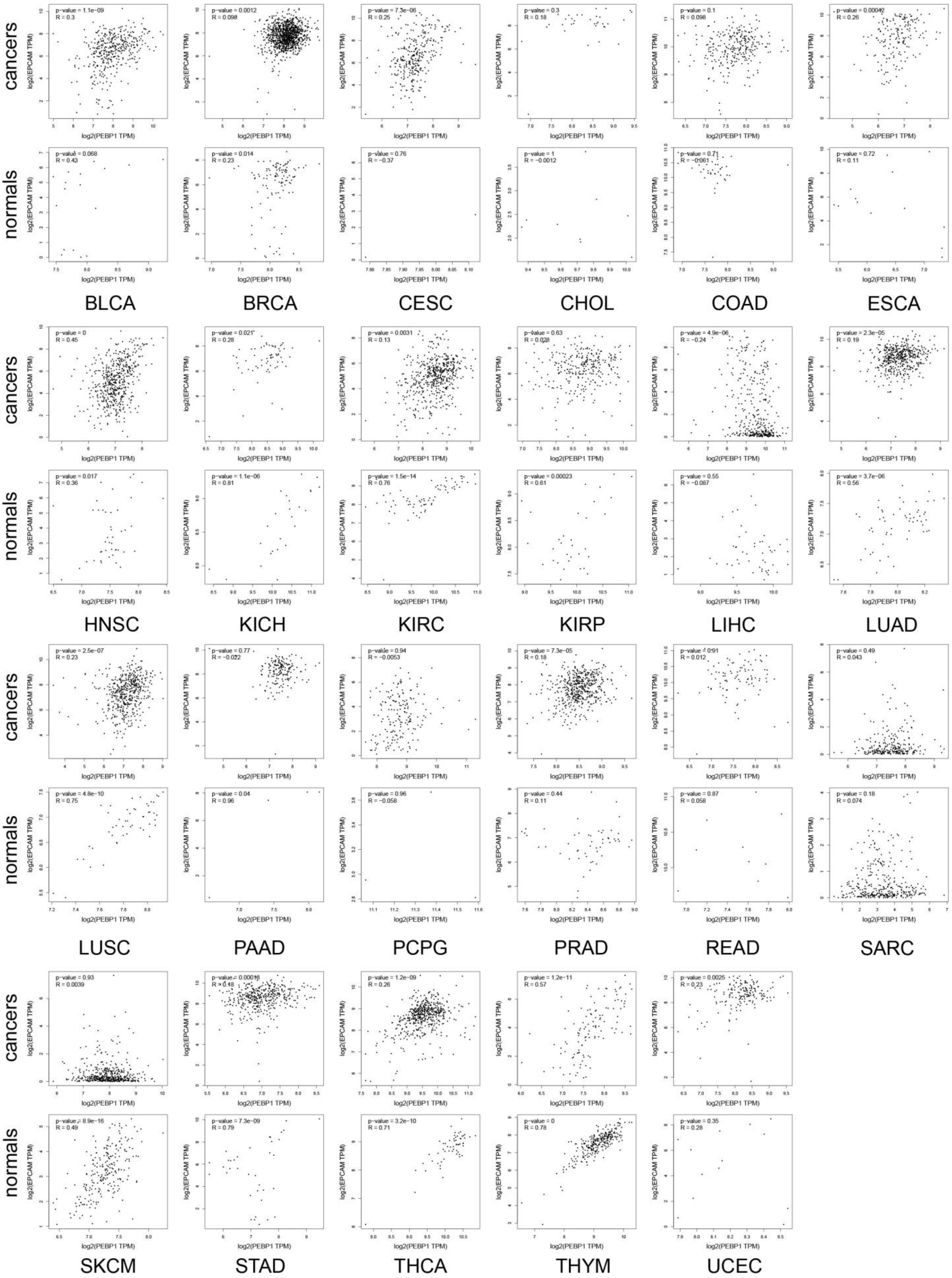
cancers
normals
log2(EPCAM TPM)
log2(PEBP1 TPM)
BLCA
p-value = 1.1e-09
R = 0.3
p-value = 0.068
R = 0.43
BRCA
p-value = 0.0012
R = 0.098
p-value = 0.014
R = 0.23
CESC
p-value = 7.3e-06
R = 0.25
p-value = 0.76
R = -0.37
CHOL
p-value = 0.3
R = 0.18
p-value = 1
R = -0.0012
COAD
p-value = 0.1
R = 0.098
p-value = 0.71
R = -0.061
ESCA
p-value = 0.00042
R = 0.26
p-value = 0.72
R = 0.11
HNSC
p-value = 0
R = 0.45
p-value = 0.017
R = 0.36
KICH
p-value = 0.021
R = 0.28
p-value = 1.1e-06
R = 0.81
KIRC
p-value = 0.0031
R = 0.13
p-value = 1.5e-14
R = 0.76
KIRP
p-value = 0.63
R = 0.028
p-value = 0.00023
R = 0.61
LIHC
p-value = 4.9e-06
R = -0.24
p-value = 0.55
R = -0.087
LUAD
p-value = 2.3e-05
R = 0.19
p-value = 3.7e-06
R = 0.56
LUSC
p-value = 2.5e-07
R = 0.23
p-value = 4.8e-10
R = 0.75
PAAD
p-value = 0.77
R = -0.022
p-value = 0.04
R = 0.96
PCPG
p-value = 0.94
R = -0.0053
p-value = 0.96
R = -0.058
PRAD
p-value = 7.3e-05
R = 0.18
p-value = 0.44
R = 0.11
READ
p-value = 0.91
R = 0.012
p-value = 0.87
R = 0.058
SARC
p-value = 0.49
R = 0.043
p-value = 0.18
R = 0.074
SKCM
p-value = 0.93
R = 0.0039
p-value = 8.9e-16
R = 0.49
STAD
p-value = 0.00018
R = 0.18
p-value = 7.3e-09
R = 0.79
THCA
p-value = 1.2e-09
R = 0.26
p-value = 3.2e-10
R = 0.71
THYM
p-value = 1.2e-11
R = 0.57
p-value = 0
R = 0.78
UCEC
p-value = 0.0025
R = 0.23
p-value = 0.35
R = 0.28

Supplementary Figure S5. RKIP vs EPCAM across normal and cancerous tissues.

Gene expression (log_2(TPM)) of the genes PEBP1 (RKIP), SNAI1 (SNAIL), VIM, CDH1 (E-Cadherin), CDH2 (N-Cadherin), EPCAM, LAMA1 and LAMB1, across 22 different types of cancer in the Cancer Genome Atlas (TCGA). BLCA, Bladder Urothelial Carcinoma; BRCA, Breast invasive carcinoma; CESC, Cervical squamous cell carcinoma and endocervical adenocarcinoma; CHOL, Cholangiocarcinoma; COAD, Colon adenocarcinoma; ESCA, Esophageal carcinoma; HNSC, Head and Neck squamous cell carcinoma; KICH, Kidney Chromophobe; KIRC, Kidney renal clear cell carcinoma; KIRP, Kidney renal papillary cell carcinoma; LIHC, Liver hepatocellular carcinoma; LUAD, Lung adenocarcinoma; LUSC, Lung squamous cell carcinoma; PAAD, Pancreatic adenocarcinoma; PCPG, Pheochromocytoma and Paraganglioma; PRAD, Prostate adenocarcinoma; READ, Rectum adenocarcinoma; SARC, Sarcoma; SKCM, Skin Cutaneous Melanoma; STAD, Stomach adenocarcinoma; THCA, Thyroid carcinoma; THYM, Thymoma; UCEC. Pair-wise Pearson's correlation between PEBP1 and EPCAM were across for each of the 22 different types of cancer and their normal tissues in the TCGA. Significance is listed for each cancer type, for each biomarker with PEBP1.

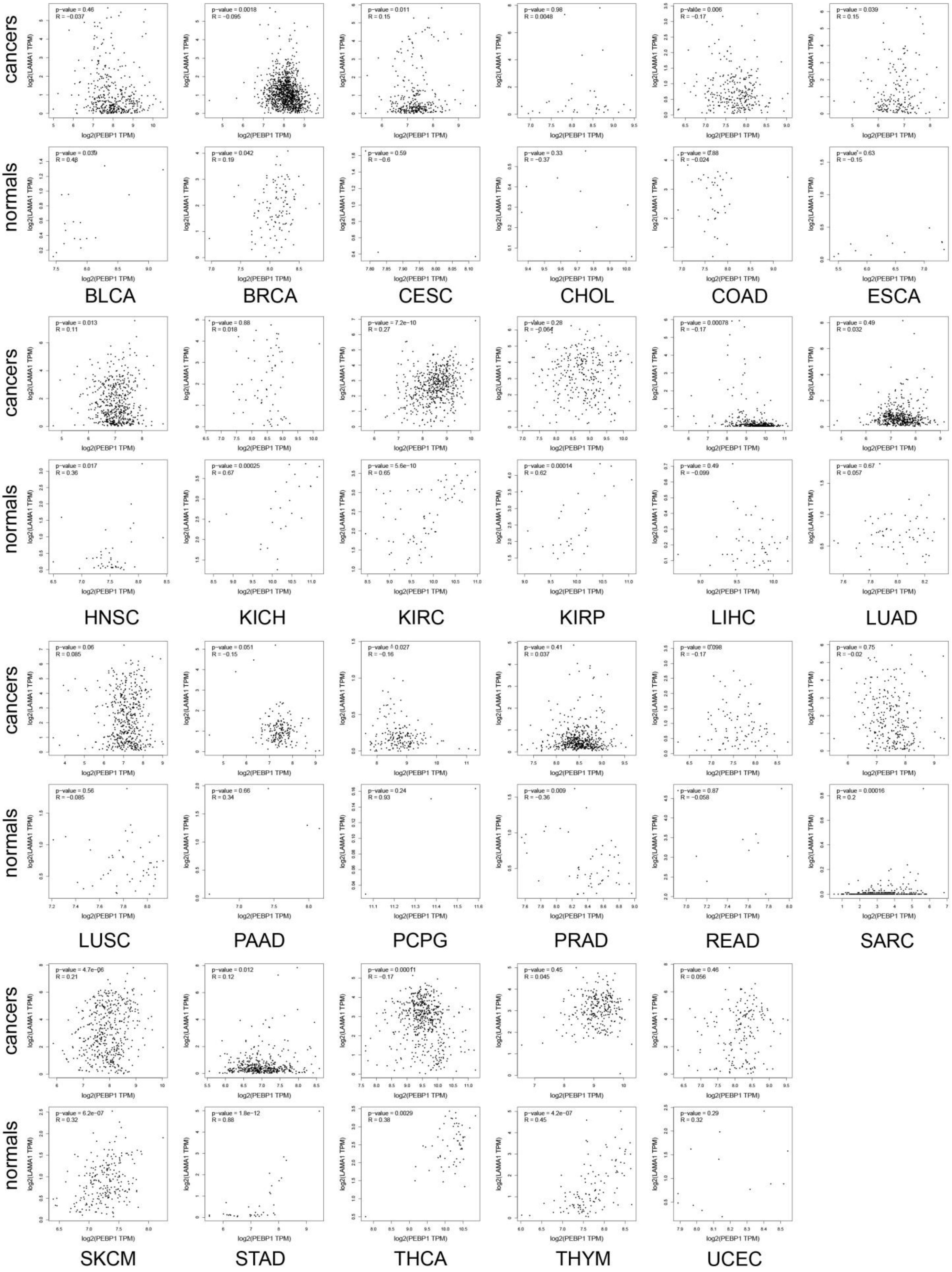
cancers
normals
p-value = 0.46
R = −0.037
p-value = 0.0018
R = −0.095
p-value = 0.011
R = 0.15
p-value = 0.98
R = 0.0048
p-value = 0.006
R = −0.17
p-value = 0.039
R = 0.15
p-value = 0.039
R = 0.48
p-value = 0.042
R = 0.19
p-value = 0.59
R = −0.6
p-value = 0.33
R = −0.37
p-value = 0.88
R = −0.024
p-value = 0.63
R = −0.15
log2(LAMA1 TPM)
log2(PEBP1 TPM)
BLCA
BRCA
CESC
CHOL
COAD
ESCA
cancers
normals
p-value = 0.013
R = 0.11
p-value = 0.88
R = 0.018
p-value = 7.2e−10
R = 0.27
p-value = 0.28
R = −0.064
p-value = 0.00078
R = −0.17
p-value = 0.49
R = 0.032
p-value = 0.017
R = 0.36
p-value = 0.00025
R = 0.67
p-value = 5.6e−10
R = 0.65
p-value = 0.00014
R = 0.62
p-value = 0.49
R = −0.099
p-value = 0.67
R = 0.057
HNSC
KICH
KIRC
KIRP
LIHC
LUAD
cancers
normals
p-value = 0.06
R = 0.085
p-value = 0.051
R = −0.15
p-value = 0.027
R = −0.16
p-value = 0.41
R = 0.037
p-value = 0.098
R = −0.17
p-value = 0.75
R = −0.02
p-value = 0.56
R = −0.085
p-value = 0.66
R = 0.34
p-value = 0.24
R = 0.93
p-value = 0.009
R = −0.36
p-value = 0.87
R = −0.058
p-value = 0.00016
R = 0.2
LUSC
PAAD
PCPG
PRAD
READ
SARC
cancers
normals
p-value = 4.7e−06
R = 0.21
p-value = 0.012
R = 0.12
p-value = 0.00011
R = −0.17
p-value = 0.45
R = 0.045
p-value = 0.46
R = 0.056
p-value = 6.2e−07
R = 0.32
p-value = 1.8e−12
R = 0.88
p-value = 0.0029
R = 0.38
p-value = 4.2e−07
R = 0.45
p-value = 0.29
R = 0.32
SKCM
STAD
THCA
THYM
UCEC

Supplementary Figure S6. RKIP vs LAMA1 across normal and cancerous tissues.

Gene expression (log_2(TPM)) of the genes PEBP1 (RKIP), SNAI1 (SNAIL), VIM, CDH1 (E-Cadherin), CDH2 (N-Cadherin), EPCAM, LAMA1 and LAMB1, across 22 different types of cancer in the Cancer Genome Atlas (TCGA). BLCA, Bladder Urothelial Carcinoma; BRCA, Breast invasive carcinoma; CESC, Cervical squamous cell carcinoma and endocervical adenocarcinoma; CHOL, Cholangiocarcinoma; COAD, Colon adenocarcinoma; ESCA, Esophageal carcinoma; HNSC, Head and Neck squamous cell carcinoma; KICH, Kidney Chromophobe; KIRC, Kidney renal clear cell carcinoma; KIRP, Kidney renal papillary cell carcinoma; LIHC, Liver hepatocellular carcinoma; LUAD, Lung adenocarcinoma; LUSC, Lung squamous cell carcinoma; PAAD, Pancreatic adenocarcinoma; PCPG, Pheochromocytoma and Paraganglioma; PRAD, Prostate adenocarcinoma; READ, Rectum adenocarcinoma; SARC, Sarcoma; SKCM, Skin Cutaneous Melanoma; STAD, Stomach adenocarcinoma; THCA, Thyroid carcinoma; THYM, Thymoma; UCEC. Pair-wise Pearson's correlation between PEBP1 and LNMA1were across for each of the 22 different types of cancer and their normal tissues in the TCGA. Significance is listed for each cancer type, for each biomarker with PEBP1.

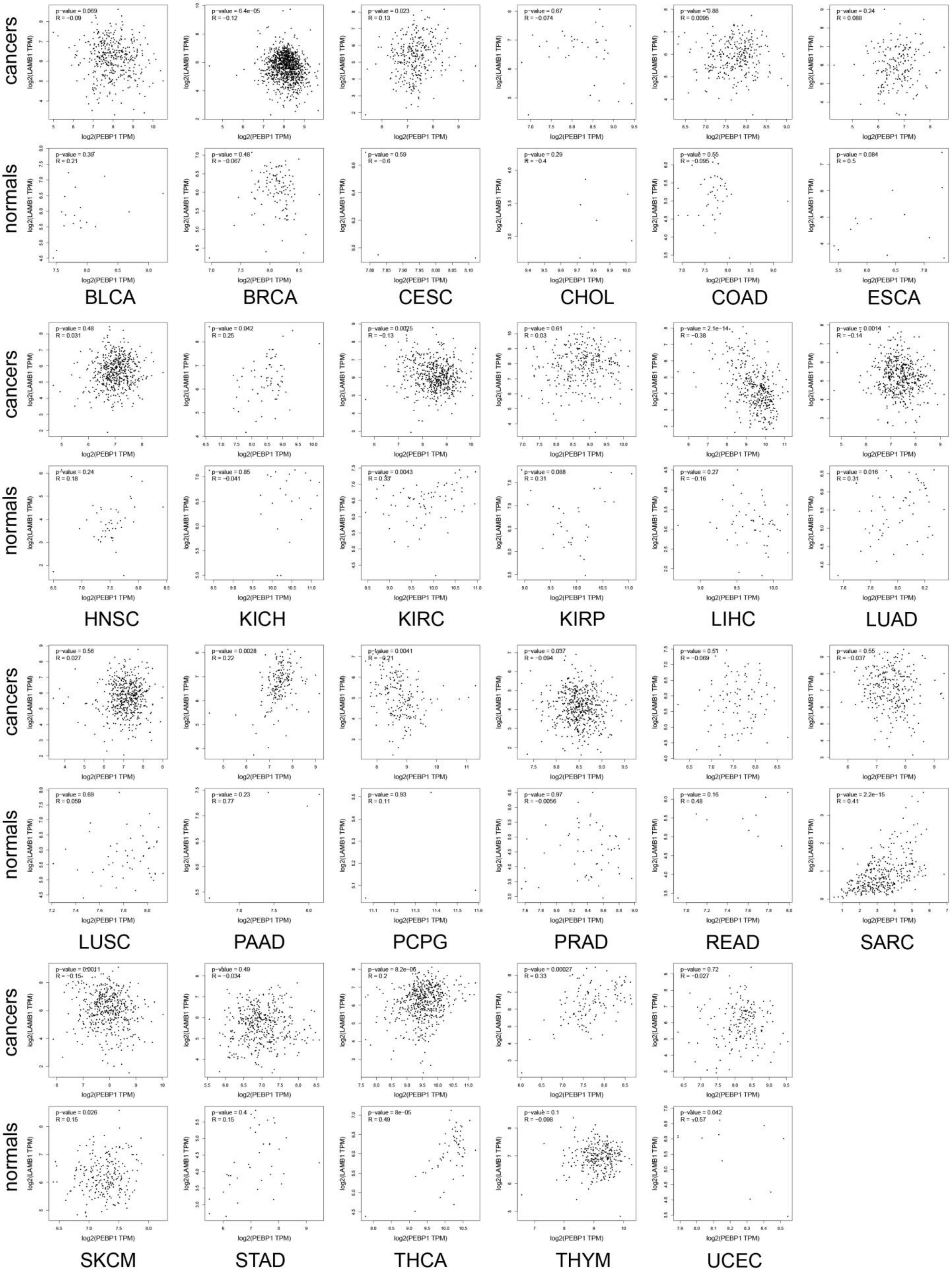
cancers
normals
BLCA
BRCA
CESC
CHOL
COAD
ESCA
HNSC
KICH
KIRC
KIRP
LIHC
LUAD
LUSC
PAAD
PCPG
PRAD
READ
SARC
SKCM
STAD
THCA
THYM
UCEC
log2(PEBP1 TPM)
log2(LAMB1 TPM)

Supplementary Figure S7. RKIP vs LAMB1 across normal and cancerous tissues.

Gene expression (log_2(TPM)) of the genes PEBP1 (RKIP), SNAI1 (SNAIL), VIM, CDH1 (E-Cadherin), CDH2 (N-Cadherin), EPCAM, LAMA1 and LAMB1, across 22 different types of cancer in the Cancer Genome Atlas (TCGA). BLCA, Bladder Urothelial Carcinoma; BRCA, Breast invasive carcinoma; CESC, Cervical squamous cell carcinoma and endocervical adenocarcinoma; CHOL, Cholangiocarcinoma; COAD, Colon adenocarcinoma; ESCA, Esophageal carcinoma; HNSC, Head and Neck squamous cell carcinoma; KICH, Kidney Chromophobe; KIRC, Kidney renal clear cell carcinoma; KIRP, Kidney renal papillary cell carcinoma; LIHC, Liver hepatocellular carcinoma; LUAD, Lung adenocarcinoma; LUSC, Lung squamous cell carcinoma; PAAD, Pancreatic adenocarcinoma; PCPG, Pheochromocytoma and Paraganglioma; PRAD, Prostate adenocarcinoma; READ, Rectum adenocarcinoma; SARC, Sarcoma; SKCM, Skin Cutaneous Melanoma; STAD, Stomach adenocarcinoma; THCA, Thyroid carcinoma; THYM, Thymoma; UCEC. Pair-wise Pearson's correlation between PEBP1 and LAMB1 were across for each of the 22 different types of cancer and their normal tissues in the TCGA. Significance is listed for each cancer type, for each biomarker with PEBP1.
